# Impact of drug loading in mesoporous silica-amorphous formulations on the physical stability of drugs with high recrystallization tendency

**DOI:** 10.1016/j.ijpx.2019.100026

**Published:** 2019-07-22

**Authors:** Rayane S.C.M.Q. Antonino, Michael Ruggiero, Zihui Song, Thais Leite Nascimento, Eliana Martins Lima, Adam Bohr, Matthias Manne Knopp, Korbinian Löbmann

**Affiliations:** a Department of Pharmacy, University of Copenhagen, Copenhagen, Denmark; b Laboratório de Nanotecnologia Farmacêutica e Sistemas de Liberação de Fármacos, Faculdade de Farmácia, Universidade Federal de Goiás – UFG, Goiânia, Goiás, Brazil; c Department of Chemistry, University of Vermont, Burlington, VT, USA; d Bioneer:FARMA, Department of Pharmacy, University of Copenhagen, Copenhagen, Denmark

**Keywords:** Mesoporous silica, Loading capacity, Differential scanning calorimetry (DSC), Amorphous

## Abstract

In this study, a method is described to determine the monolayer loading capacity (MLC) of the drugs naproxen and ibuprofen, both having high recrystallization tendencies, in mesoporous silica (MS), a well known carrier that is able to stabilize the amorphous form of a drug. The stabilization has been suggested to be due to direct absorption of the drug molecules onto the MS surface, i.e. the drug monolayer. In addition, drug that is not in direct contact with MS surface can fill the pores up to its pore filling capacity (PFC) and is potentially stabilized by confinement due to the pore size being smaller than a crystal nuclei. For drugs with high recrystallization tendencies, any drug outside the pores crystallizes due to its poor physical stability. The drug monolayer does not contribute to the glass transition temperature (*T_g_*) in the DSC, however, the confined amorphous drug above MLC has a *T_g_* and the heat capacity (Δ*C*_p_) over the *T_g_* increases with an increasing fraction of confined amorphous drug. Hence, several drug loading values above the MLC were investigated towards the presence of a *T_g_* and Δ*C*_p_ using differential scanning calorimetry (DSC). A linear correlation between the amount of confined amorphous drug and its Δ*C*_p_ was identified for the mixtures between the MLC and PFC. By subsequent extrapolation to zero Δ*C*_p_ the experimental MLC could be determined. Using theoretical density functional theory (DFT) and *ab initio* Molecular Dynamics (AIMD), the binding energies for the monolayer suggested that the monolayer in fact is thermodynamically more favorable than the crystalline form, whereas the confined amorphous form is thermodynamically less favorable. Consequently, a physical stability study showed that the confined amorphous drugs above the MLC were thermodynamically unstable and consequently flowing out of the pores in order to crystallize, whereas the monolayer remained physically stable.

## Graphical abstract

**Figure:**
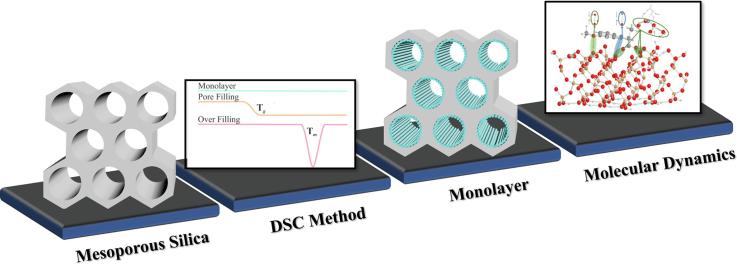

## 1 Introduction

Amorphous formulations are one of the most efficient ways of improving bioavailability in an era of drug discovery where a large percentage of new molecules have solubility-limited dissolution rates (Riikonen et al., 2018, Sayed et al., 2018). In this context, mesoporous silica (MS), having small pores (e.g., pore diameter between 2 and 50 nm) and large specific surface areas (e.g., often greater than 300 m^2^/g) (Andersson et al., 2004), have received quite some attention, due to their ability to stabilize the amorphous form of a drug within their mesopores (Kumar et al., 2014, Laitinen et al., 2013, Rouquerol et al., 1994).

Inhibition of drug crystallization through adsorption to a MS has generally been explained by two responsible mechanisms: i) molecular interactions (e.g., hydrogen bonding) between functional groups of the drug molecules and the surface of the MS, and ii) confinement and spatial separation of the drug in the pores of MS, since the diameter of the mesopores is smaller than a critical crystalline nuclei of the drug (Azaïs et al., 2006, Rengarajan et al., 2008). With respect to i), the large MS surface area provides additional surface free energy, and it has been suggested that the adsorption of the drug in the amorphous form is actually thermodynamically favorable because of the lower free energy state than the crystalline drug (Andersson et al., 2004, Qian and Bogner, 2011, Qian and Bogner, 2012). When all binding sites on the MS surface are occupied by drug molecules and an excess amount of drug is present in the system, it cannot be in direct contact with the MS surface anymore, and it will start to form additional layers on top of the initial drug monolayer (Genina et al., 2018, Hempel et al., 2018). In this case, the drug will start filling up the pores and this excess amount of amorphous drug may be stabilized by being physically restrained from crystallization, in the case of ii). Thus, the surface area and pore volume of a given MS influence the loading capacity of a given drug (Bavnhøj et al., 2019, Yani et al., 2016).

Accordingly, the loading capacity of a drug in a MS can be differentiated into two different classes, i.e. the drug in direct contact with the MS surface forming a drug monolayer and any excess drug filling up the pores. The former loading limit is dependent on the available surface area of the MS and is referred to as monolayer loading capacity (MLC). The latter is dependent on the pore volume of the MS and is referred to as pore filling capacity (PFC). Any further addition of drug will result in an overloading, i.e. drug being present outside of the pores (Bavnhøj et al., 2019, Choudhari et al., 2014).

The MLC can be determined experimentally by a differential scanning calorimetry (DSC) based method recently introduced by Hempel et al. (2018). This method is based on deliberately overloading the MS with the drug upon melting and quenching (using drug loadings of 50–90 wt%), and subsequently determining the heat capacity change (Δ*C*_p_) over the glass transition temperature (*T_g_*) of the excess drug (i.e. excess to the monolayer) upon reheating. Since the monolayer is not contributing to the *T_g_* signal in the DSC (Shen et al., 2010), the MLC can be obtained by extrapolating the Δ*C*_p_ values for the different drug loading values to zero (x-intercept). Since the method relies on the presence of an excess amorphous phase providing a *T_g_* signal, it works well for drugs with good or medium glass forming ability (GFA). Generally, drugs can be classified into three classes of GFA according to their tendency to crystallize from the undercooled melt (Baird et al., 2010), i.e. based on the presence/absence of observable crystallization during a heat-cool-heat cycle using DSC. Briefly, class I drugs are classified as poor glass formers and crystallize upon cooling of the melt, class II are medium glass formers that do not crystallize upon cooling from the melt but upon reheating above their *T_g_* and class III are good glass formers that neither crystallize upon cooling and reheating (Avramov et al., 2003). In other words, the approach of Hempel et al. (2018) is not feasible for drugs with poor GFA since these drugs would crystallize outside the MS pores (at least above PFC), making a meaningful determination of the Δ*C*_p_ over the *T_g_* of the excess drug at high drug loadings above 50 wt% not possible. On the other hand, it has been suggested that at concentrations above the MLC but below the PFC, the drug will be constrained within the pores and a crystallization cannot occur within the pores due to the pore diameter being smaller than a crystal nuclei (Qian and Bogner, 2012).

Consequently, the aim of this study was to investigate whether the MLC of a class I drug with poor GFA, namely naproxen, can experimentally be determined by extending the drug-MS ratios to lower drug loading to cover the region between MLC and PFC. Furthermore, the impact of different degrees of drug loading, i.e. monolayer, pore filling and overfilling, on the physical stability of such a system was studied and compared to ibuprofen, a drug with good GFA (class III). Lastly, the impact of drug loading upon storage below and above the *T_g_* was investigated, in particular with a focus on the amorphous (in)stability of the confined drug above MLC but below PFC, both for poor and good glass formers.

## 2 Methods and materials

### 2.1 Materials

Naproxen (NAP; *M*_w_ = 230.26 g/mol, minimal projection area 34.77 Å^2^, maximal projection area 72.19 Å^2^, molecular density 1.081 g/cm^3^) and ibuprofen (IBU; *M*_w_ = 206.28 g/mol, minimal projection area 35.44 Å^2^, maximal projection area 64.57 Å^2^, molecular density 0.974 g/cm^3^) were purchased from Fagron (Barsbüttel, Germany). Syloid® 72 FP (SYL; average pore diameter 10 nm, pore volume 1.20 cm^3^/g, surface area 350 m^2^/g) was received as a generous gift from Grace GmbH (Worms, Germany). All chemicals were used as received.

### 2.2 Experimental MLC determination

Based on a method proposed by Hempel et al. (2018), the MLC was determined from physical mixtures of the crystalline drugs with SYL. Physical mixtures of drug and SYL (15–100 wt% drug for IBU and 10–100 wt% drug for NAP) were prepared by weighing in a total of 200 mg of the material followed by gentle mixing using a mortar and pestle. The mixing procedure was repeated three times in order to ensure proper mixing before the powder was collected and stored in an airtight container at room temperature until use. The thermal properties of the samples were analyzed using a Discovery DSC from TA Instruments (New Castle, DE, USA). The physical mixtures of the IBU samples (~5 mg) and NAP samples (~14 mg) were analyzed in Tzero aluminum pans with a perforated lid under 50 mL/min nitrogen gas purge. The *T*_g_ (midpoint) and the heat capacity change over the glass transition (Δ*C*_p_) were determined using the TA Instruments TRIOS (version 4.1.1) software.

For the determination of the MLC of IBU in SYL, the physical mixtures were exposed to a heat-cool-heat cycle using standard DSC. The physical mixtures were first annealed at ~5 °C above the melting point (*T_m_*) of the drug for 5 min to ensure complete fusion of the drug into the pores and then quench cooled at a ballistic rate (maximum cooling rate of the instrument) to −80 °C. The samples were subsequently heated at a rate of 20 °C/min to 30 °C above the *T_m_* of the drug. Each experiment was conducted in duplicate.

For the determination of the MLC of NAP in SYL, the physical mixtures were exposed to a heat-cool-heat cycle using modulated DSC. The physical mixtures were first annealed at ~5 °C above the *T_m_* of the drug for 5 min to ensure complete fusion of the drug into the pores and then cooled to −80 °C at 10 °C/min. Subsequently, a modulated temperature DSC was used to determine the *T_g_* and Δ*C*_p_ (J/g °C) due to the higher sensitivity compared to standard DSC*.* The samples were analyzed from −80 °C to 80 °C at a heating rate of 2 °C/min with an underlying modulated temperature amplitude of 1.0 °C and a period of 50 s. The *T_g_* and Δ*C*_p_ were determined from the reversing heat flow signal. Each experiment was conducted in duplicate.

The MLC was determined by a linear fitting of Δ*C*_p_ as a function of drug loading in the physical mixtures. For IBU and NAP, the linear fitting was performed on the drug loadings from 30 to 100 (wt%) and 20–50 (wt%), respectively. The experimental MLC is then obtained from the x-intercept of the trendline. Furthermore, the prediction interval with the upper and lower limits based on a 95% confidence interval were determined considering each replicate of Δ*C*_p_ as individual data point.

### 2.3 Theoretical determination of the MLC and PFC

The theoretical MLC and PFC were based on a previous publication by Bavnhøj et al. (2019). Briefly, the theoretical MLC is based on the minimum projected surface area of the drug molecules and was calculated from Eqs. (1), (2):

(1) $${\mathrm{MLC}}_{w}=\frac{A_{\mathrm{MS}}∙M_{w(drug)}}{A_{\mathrm{drug}}∙N_{A}}$$

where *A*_MS_ is the surface area of the respective MS (m^2^/g), *A*_drug_ is the minimal or maximal projection (surface) area of the respective drug (m^2^/molecule) estimated using MarvinSketch version 18.12 from ChemAxon (Budapest, Hungary), *N_A_* is the Avogadro constant (6.022·10^23^ mol^−1^) and *M_w_*_(drug)_ is the molecular weight (g/mol) of the respective drug. Eq. (1) calculates the MLC as *w_drug_/w_MS_* (*MLC_w_*). The theoretical MLC as wt% of the entire formulation, i.e. *w_drug_/(w_drug_ + w_MS_)*, was calculated using Eq. (2):

(2) $${\mathrm{MLC}}_{\mathrm{wt}\%}=\frac{{\mathrm{MLC}}_{w}}{1+{\mathrm{MLC}}_{w}}∙100\%$$

The theoretical PFC was calculated based on the amorphous/molecular densities of the drugs and pore volume of the MS, according to the Eq. (3):

(3) $$\mathrm{PFC}=\frac{V_{\mathrm{MSpore}}∙\rho_{\mathrm{drug}}}{1+V_{\mathrm{MSpore}}∙\rho_{\mathrm{drug}}}∙100\%$$

where *V*_MS pore_ is the pore volume of the MS (cm^3^/g) and *ρ*_drug_ is the molecular density of the drug estimated using MarvinSketch version 18.12 from ChemAxon (Budapest, Hungary). The PFC includes the drug in the monolayer as well as the excess drug confined by the pores.

### 2.4 Theoretical *ab initio* molecular dynamics (AIMD) and density functional theory (DFT) simulations

The CP2k software package was used for all AIMD simulations, which incorporated three-dimensional periodic boundary conditions (Hutter et al., 2014, VandeVondele et al., 2005). With the AIMD method, the atomic dynamics are allowed to evolve in time according to Newton’s equations of motion (i.e. *F = ma*), with the distinction between classical molecular dynamics being that the forces in AIMD are recomputed using quantum-mechanical simulations at each discrete timestep in the simulation. Thus, the AIMD technique enables the inclusion of temperature, providing a meaningful description of the structures and dynamics of materials at a high-level of theoretical accuracy. The simulations made use of the Perdew-Burke-Ernzerhof (PBE) density functional (Perdew et al., 1996) coupled with the dispersion correction of Grimme (Grimme-D3 (Grimme et al., 2010, Grimme et al., 2011). The electronic wavefunctions were represented using the double-zeta DZVP basis set (VandeVondele and Hutter, 2007). Simulations were performed within the canonical ensemble (NVT), with the temperature maintained at 200 K using a Nose-Hoover chain thermostat (Martyna et al., 1992, Nosé, 1984, Nosé, 2002). The initial model involved loading the porous void with drug molecules to a loading limit of 10% less than the crystallographic density of the respective solids. The surface functionalization was set to an average OH-distribution of approximately 4.5 OH nm^−1^ to be as similar to the physically used SYL surface as possible. The CRYSTAL17 (Dovesi et al., 2018) software package was used for static DFT simulations to represent a two-dimensional periodic surface (periodic along the x and y axes corresponding to an infinite surface slab) with a single API molecule in the simulation cell. In order to match the AIMD simulations, parameters were kept as similar as possible. The D3-corrected PBE functional was coupled with the def2-SVP (Weigend and Ahlrichs, 2005) basis set for all atoms. Optimizations were performed at an effective temperature of 0 K to extract the fundamentally stable binding geometry of the adsorbed molecules.

### 2.5 Physical stability study

Physical mixtures of drug and SYL (15–60 wt% drug in 5 wt% increments for IBU and 10–60 wt% drug in 5 wt% increments for NAP) were prepared as described above and subsequently molten in a UF55 oven from Memmert (Schwabach, Germany) at 5 °C above *T_m_* of the respective drug for 5 min. Subsequently, the mixture was removed from the oven, quench cooled to room temperature and gently mixed using a mortar and pestle. The procedure was repeated once more to ensure that the mixture was homogeneous and the solid-state characteristics of the powder samples were then analyzed using an X’Pert Pro diffractometer from PANalytical (Almelo, the Netherlands) using CuKα radiation (λ = 1.5406 Å) at 45 kV and 40 mA. The freshly prepared samples were analyzed directly after preparation and after storage for 4 weeks under ambient conditions and at −80 °C in a closed container. Approximately 3 mg of sample was placed on aluminum plates and measured over the angular range of 5–30° 2θ at a scanning rate of 4° 2θ/min and resolution of 0.001° 2θ. Results were analyzed using the X’Pert Data Viewer (version 1.2) software.

## 3 Results and discussion

As mentioned in the introduction, it has previously been shown that a DSC based method can be used to determine the MLC of a drug with medium and good GFA in MS (Hempel et al., 2018). IBU is such a good glass former, however, NAP is a poor glass former and recrystallizes quickly already in the quenching step during the DSC run (Baird et al., 2010, Blaabjerg et al., 2017). Hence, any excess NAP outside of the pores would crystallize, which makes a meaningful determination of the Δ*C*_p_ over the *T_g_* of the excess drug at a high drug loading above 50 wt% not possible. However, since the drug will be constrained within the pores at concentrations above the MLC but below the PFC, a crystallization can in theory not occur within the pores due to the pore diameter being smaller than a crystal nuclei (Qian and Bogner, 2012). For this purpose, we have extended the drug-MS ratios to lower a drug loading to cover the region between MLC and PFC in this study, i.e. 15–100 wt% for IBU and 10–100 wt% for NAP. It is suggested that such an approach will potentially allow the determination of Δ*C*_p_ values also for compounds with poor GFA, such as NAP (at least for a drug loading ranging between MLC and PFC), allowing for a determination of their MLC. In this case, the confined drug (pore filling) above the MLC would remain amorphous, resulting in a *T_g_* and Δ*C*_p_ signal in the DSC, while the drug outside the MS pores would crystallize.

**Fig. 1:**
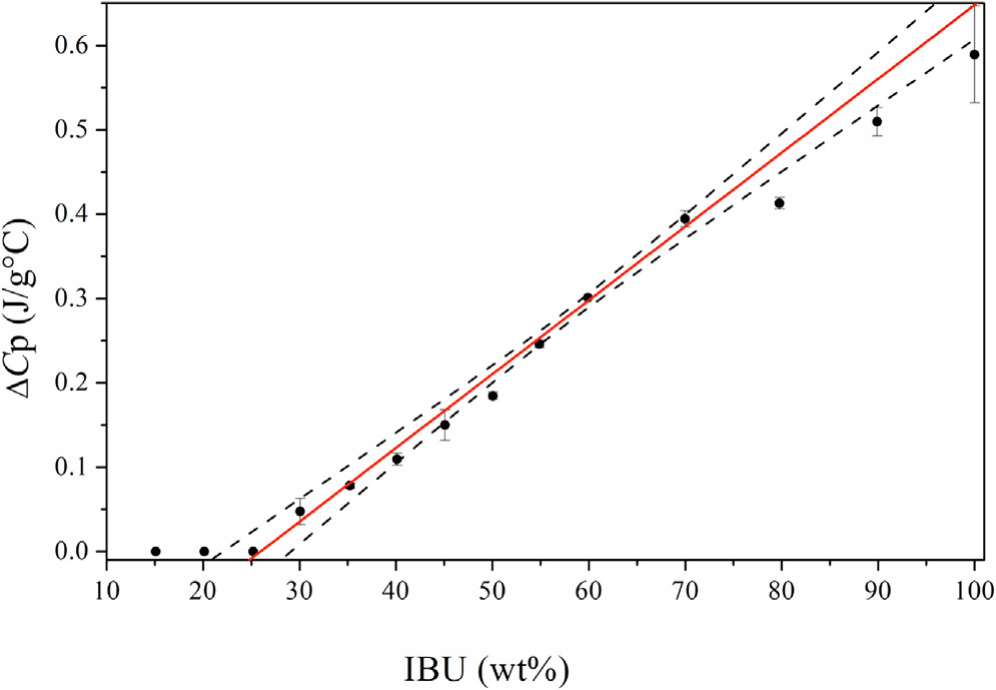
Experimentally obtained Δ*C*_p_ (J/g °C) values over *T_g_* as a function of IBU (wt%) loaded on SYL as well as their linear extrapolation between 30 and 100 wt% in SYL, r^2^ = 0.99. The 95% confidence interval is represented in the dashed lines.

For IBU-SYL, the experimentally determined MLC at zero Δ*C*_p_ (x-intercept) was found to be 26.6 wt% with a prediction interval from 22.6 to 29.9 wt% (Fig. 1), which was supported by the absence of a *T_g_* at a drug load of 25 wt% or below, suggesting that all drug was present as a monolayer for these drug loadings. The theoretical MLC based on minimum and maximum projected surface area was calculated at 25.3 and 15.9 wt%, respectively. The close agreement of the experimental MLC and the theoretical MLC based on minimum projected surface area suggest that the IBU molecules adsorb densely to the MS surface, i.e. occupying as little surface area as possible. The theoretical PFC was calculated to be 53.9 wt%. Since IBU is a good glass former, the amorphous fraction of the drug from the pore filling (between MLC and PFC) and outside the pores (overfilling), both contribute to the Δ*C*_p_, resulting in a linear increase in the Δ*C*_p_ values over the entire sample set above MLC.

**Fig. 2:**
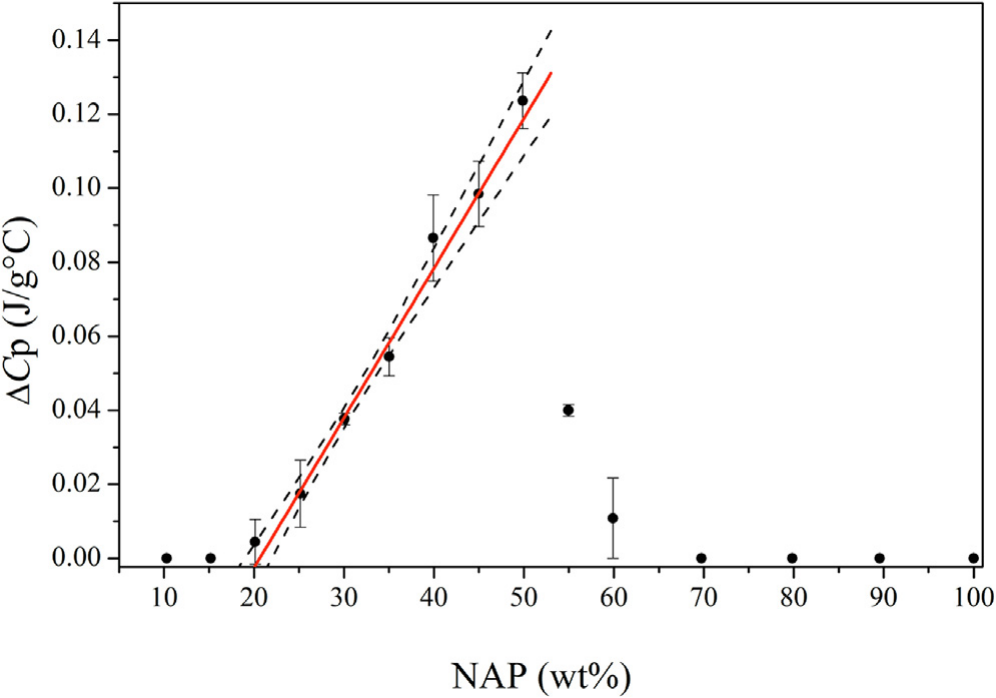
Experimentally obtained Δ*C*_p_ (J/g °C) values over *T_g_* as a function of NAP (wt%) loaded on SYL as well as their linear extrapolation between 20 and 50 wt% in SYL, r^2^ = 0.99. The 95% confidence interval is represented in the dashed lines.

For NAP-SYL, a Δ*C*_p_ value may in theory only be obtained for the amorphous drug fraction between MLC and PFC. In other words, it is expected that a Δ*C*_p_ value is detectable for any drug loading above MLC and below PFC. For drug loadings above PFC, one would assume that the pores are completely filled with drug and additionally excess drug would be on the outside of the pores. Since NAP is a poor glass former, the drug outside the pores should crystallize, however, the drug constrained inside the pores could be amorphous and should be detectable. From Fig. 2, it can be seen that below 20 wt% drug loading, no *T_g_*s or Δ*C*_p_ values were obtained. For the samples with drug loadings between 20 and 60 wt%, indeed Δ*C*_p_ values could be identified and a linear increase of Δ*C*_p_ was obtained for the samples between 20 and 50 wt%, suggesting that amorphous NAP is indeed filling up the pores. When using the Δ*C*_p_ values for the samples between 20 and 50 wt%, an experimental MLC at zero Δ*C*_p_ (x-intercept) was determined at 20.6 wt% with a prediction interval from 19.1 to 22.2 wt%. The theoretical MLC based on the minimum and maximum projected surface area for NAP were calculated as 27.8 and 15.6 wt%, respectively. Since the experimental MLC lies in between these two theoretical MLC values, it is suggested that the NAP molecules are occupying a larger surface area on the MS surface than calculated from the minimal projected surface area of NAP. Hence, in contrast to IBU, the NAP molecules do not adsorb as densely to the MS surface.

Interestingly, the Δ*C*_p_ decreases with increasing NAP load (55 and 60 wt%) and then disappears for samples with drug loadings >70 wt%, suggesting that a part or all of the drug above MLC is crystallizing during the timeframe of the heat-cool-heat cycle in the DSC. In other words, this suggests that the excess drug (above MLC and below PFC) must rather flow out of the pores and crystallize outside of the pores than remaining confined within the pores. To understand this finding one needs to consider that i) only the monolayer drug is thermodynamically favorable compared to the crystalline drug (Qian and Bogner, 2012), ii) the confined drug not in the monolayer is purely prevented from crystallization by the pore size being smaller than a crystal nuclei (Qian and Bogner, 2012) and iii) the crystalline form of the drug is thermodynamically more stable than the pure amorphous form (Qian and Bogner, 2011) also when confined within the pores (above MLC and below PFC). The findings suggest that if there is a possibility for the drug to crystallize (drug loading above PFC), it will crystallize quickly and any drug confined in the pores will flow out of the pores and also crystallize. One may think of the drug crystallizing outside of the pores acting as seed for further crystal growth, dragging the confined drug out of the pores since it would be able to change into a thermodynamically more favorable form. This is further supported by the low *T_g_* of NAP, its poor GFA and its high, inherent tendency to crystallize. For the samples with a drug loading of 70 wt% and above, this effect becomes so pronounced that all of the confined drug (except the monolayer drug) recrystallizes on the outside of the pores. For the samples containing 55 and 60 wt% drug, i.e. close to the PFC, this effect is only partial, and some amorphous drug can remain within the pores during the timeframe of the DSC run, hence, contributing to the *T_g_* and Δ*C*_p_ signal in the DSC measurement.

In order to investigate the underlying energetics driving intermolecular drug-surface and amorphous drug-drug interactions, the binding energies of the various phases of the drugs were determined using solid-state DFT and AIMD simulations. For these simulations, two distinct models were generated and used, the first was a physical representation of the studied systems, consisting of a MS model, which was loaded with drug to a density (within the pore) of 10% less than the crystallographic density of the respective drug molecules. The second was a two-dimensional periodic simulation where a drug molecule was fully docked to the surface and allowed to relax in order to strictly determine the potential of the drug-monolayer binding interaction. The results of the two analyses for the adsorbed drug were very similar; the ‘physical’ model yields a relative drug-monolayer binding energy (compared to the crystalline drug binding energy) for IBU of −32.93 kJ mol^−1^, while the drug-surface 2D model yields a value of −31.98 kJ mol^−1^. The results of the 2D monolayer model are provided in Table 1 for both of the studied materials.

**Table 1:** Binding energies and relative binding energies (ΔE, compared to the crystalline binding energies) for NAP and IBU for the monolayer and amorphous phases of the two materials calculated from DFT simulations. All energies are in units of kJ mol^−1^ molecule^−1^.

	Naproxen		Ibuprofen	
	Binding Energy	ΔE	Binding Energy	ΔE
Crystal	−235.28	–	−136.61	–
Monolayer	−307.90	−72.62	−169.54	−32.93
Amorphous	−167.14	+68.14	−41.79	+94.82

**Fig. 3:**
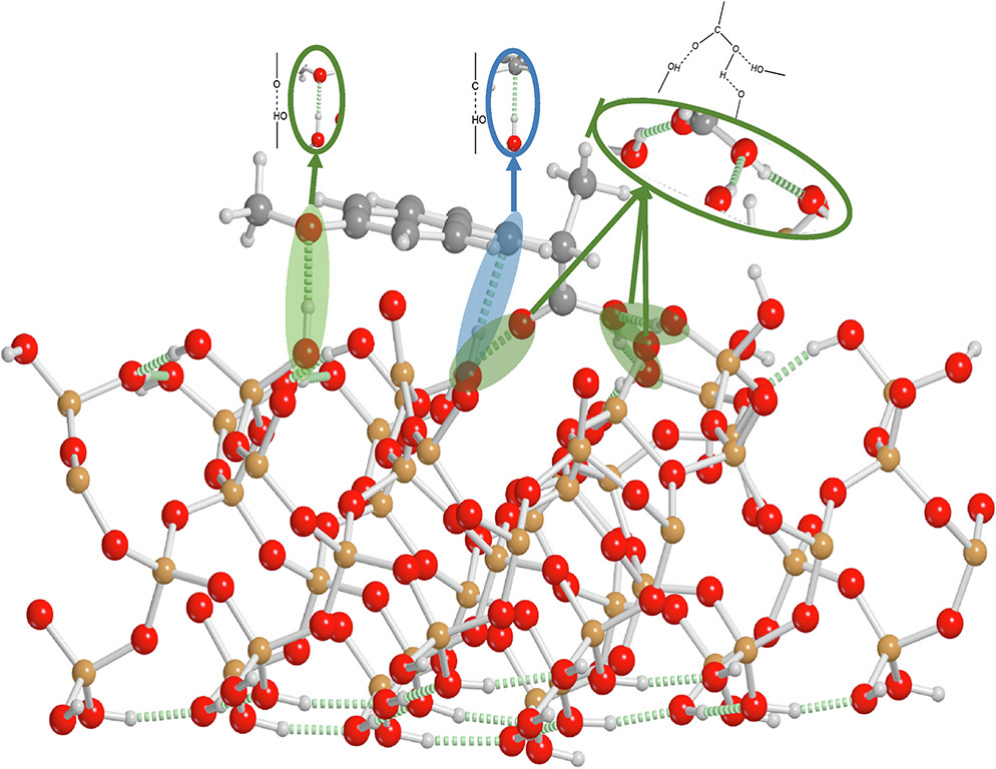
Model of the NAP-SYL surface showing traditional hydrogen bonding (green circles), and non-traditional C***H–O hydrogen bond formation (blue circle).

In both cases, the energetics point to the previously observed effect that the drug-monolayer interaction is more stable than in the corresponding crystalline form. Moreover, a fully amorphous model yields net positive relative binding energies for the two materials, in line with experimental expectations of the instability of the amorphous form and the preference for crystallization. The simulations accurately confirm that monolayer adsorbed drugs are more stable than the corresponding crystalline counterparts, and a structural investigation provides some rationale for this effect. It is important to note that the binding energy is defined as

(4) $$E_{\mathrm{binding}}=E_{\mathrm{drug}+surface}-\left(E_{\mathrm{drug}}+E_{\mathrm{surface}}\right)$$

such that the binding energy is not directly related to either the individual structures of the surface or drug on its own. However, the lack of a fully periodic and well-ordered crystalline structure indirectly improves the binding energy of the drug-surface interaction by enabling the drug molecules to adopt a conformation that maximizes each individual interaction while at the same time allowing for conformational freedom to adopt a more optimal molecular structure. For example, in the NAP system, the crystalline interactions are dominated by hydrogen bonding interactions between the carboxyl groups, with some weak London dispersion forces between the hydrophobic core of the molecule, and the individual molecular conformations are relatively stained in order to maximize and balance intermolecular interactions with the conformational strain. However, in the NAP-SYL system, the lack of well-defined order and the nature of the MS surface allows the NAP conformation more freedom to adopt a favorable structure, with the surface subsequently adapting its geometry to accommodate the NAP molecules. For example, hydrogen bonding interactions are maximized in the adsorbed system, including the carboxylic acid and ether groups of NAP (Fig. 3), as there are more hydrogen bond donors on the MS surface than what are present in crystalline NAP. Furthermore, non-traditional interactions are present, for example weak C***H–O hydrogen bonds, which do not provide enough of an energetic stabilization to be observed in crystalline NAP, and are readily present due to the overabundance of hydrogen bond donors on the MS surface. Additionally, the increased dispersion forces due to the highly polar surface, and the more optimal binding geometry of the drugs all combine to drive the binding of the drugs and increased stability of the drug-SYL materials.

Finally, the simulations provide a rationale for the theoretical determination of the MLC for NAP previously described. As described above, the experimental MLC value for NAP falls in between the predicted values using the minimum and maximum projected surface areas of the individual molecules. Since the projected surface areas of NAP in these calculations do not account for how the molecules bind to the surface, this observation is readily explained by investigating the binding geometry of the drug molecules with respect to the surface (Fig. 3). Due to the maximized hydrogen bonding of the NAP molecules on the MS surface, the drug molecules are not oriented completely orthogonal to the SYL surface, but are slightly rotated. Considering the hydrogen bonding pattern of the NAP molecules, the DFT calculations allow to project a minimum and maximum surface area of 51.33 Å^2^ and 65.37 Å^2^. These corrected values were used in Eqs. (1), (2), and the theoretical MLC based on the minimum and maximum projected surface area for NAP are now 20.7 and 17.0 wt%, respectively. The close agreement of the experimental MLC (20.6 wt%) to the corrected theoretical MLC based on minimum projected surface area suggest that the NAP molecules indeed are fulfilling their maximum hydrogen bonding possibilities and subsequently adsorb densely to the MS surface, i.e. occupying as little surface area as possible.

In order to investigate the effect of drug loading on the physical stability, drug-SYL systems were prepared with a drug loading between 15 and 60 wt% for IBU and 10–60 wt% for NAP and analyzed using XRPD directly after preparation and after 4 weeks of storage at −80 °C (below the *T_g_* of the drugs) or under ambient conditions (above the *T_g_* of the drugs). It was assumed that below the MLC, these systems would be thermodynamically stable and between the MLC and PFC, they would be sterically stabilized (physically stable) by the drug confinement. Storing the samples below and above their respective *T_g_* would potentially further contribute to stabilization or destabilization of the confined amorphous drug, since above the *T_g_*, the drugs may possess enough mobility to leak from the pores and crystallize on the outside of the pores. The pure amorphous IBU and NAP are known to be highly unstable and rapidly recrystallize after preparation, mainly because of their very low *T_g_* of −45 °C and 5 °C, respectively (Blaabjerg et al., 2017).

**Fig. 4:**
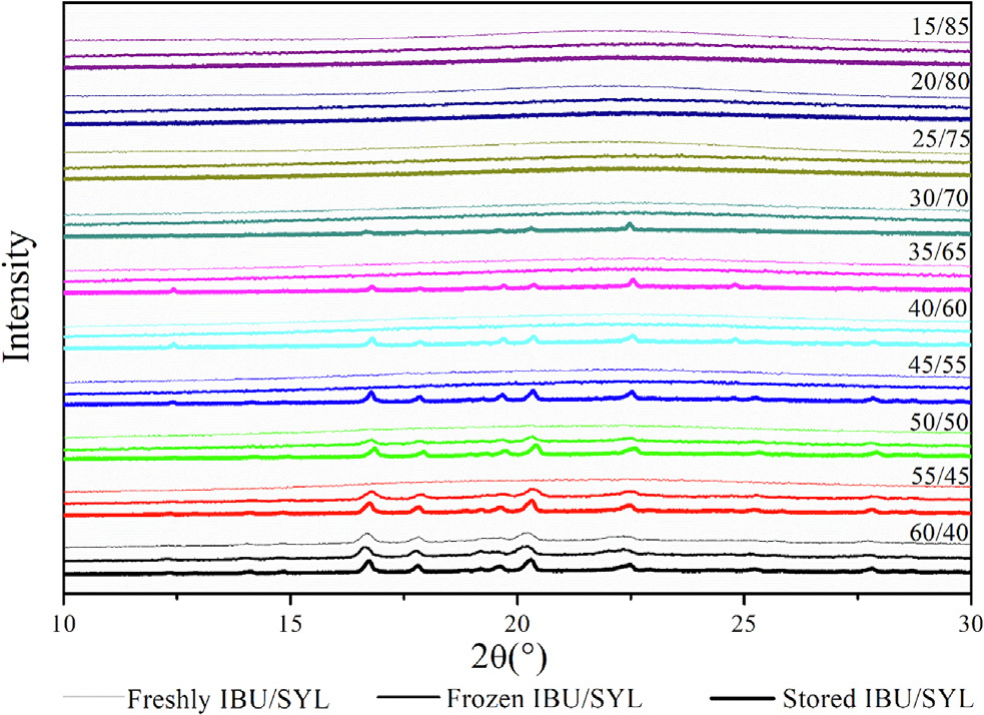
X-rays diffractograms of IBU/SYL systems with different drug loadings freshly prepared (top diffractogram within a given drug loading XRPD set) and stored for 4 weeks either at −80 °C (middle diffractogram within a given drug loading XRPD set) or under ambient conditions (bottom diffractogram within a given drug loading XRPD set).

The XRPD diffractograms for the freshly prepared IBU-SYL systems showed that all drug loadings except 60 wt% were fully amorphous systems (Fig. 4). This finding confirms that IBU was completely loaded into the pores of SYL up to 55 wt%. Since the theoretical PFC for IBU was found to be 53.9 wt%, it was expected that some crystallinity was observed for the 60 wt%, being above the PFC, and due to the poor physical stability of the pure amorphous IBU. The experimental MLC was 26.6 wt%, suggesting that samples with drug loadings ≤25 wt% are below the MLC and samples between 30 and 55 wt% represent those with pore filling. Upon storage, it was observed that the drug loadings below the MLC remained amorphous regardless of the storage conditions. However, for the samples between 30 and 55 wt% drug loading, Bragg peaks characteristic of crystalline IBU could be identified depending on the storage conditions. When storing the samples above the *T_g_* of IBU (ambient conditions), all samples above the MLC recrystallized, whereas when storing below its *T_g_* (at −80 °C) only the two samples close to the PFC, i.e. 50 and 55 (wt%), showed recrystallization (Fig. 4).

**Fig. 5:**
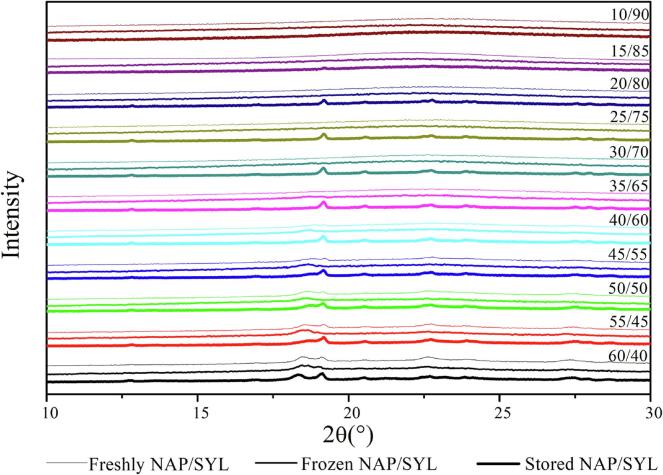
X-rays diffractograms of NAP/SYL systems with different drug loadings freshly prepared (top diffractogram within a given drug loading XRPD set) and stored for 4 weeks either at −80 °C (middle diffractogram within a given drug loading XRPD set) or under ambient conditions (bottom diffractogram within a given drug loading XRPD set).

The XRPD patterns for the freshly prepared NAP-SYL systems show that fully amorphous systems can be prepared up to 35 wt% drug loading (Fig. 5). This suggests that amorphous NAP-SYL systems can be prepared above the experimental MLC (20.6 wt%) but not entirely up to its PFC (56.5 wt%) as seen for IBU. Furthermore, given the linear increase of the Δ*C*_p_ up to a drug loading of 50 wt% in the DSC (see above), one may have expected that it would be possible to prepare fully amorphous samples also up to a drug loading of 50 wt%. However, the findings indicate that at loadings above 35 wt%, at least parts of the confined drug leaked out of the pores and crystallized as suggested above. Due to the longer experimental time frame from preparation to XRPD analysis compared to the rather short heat-cool-heat cycle during the DSC runs, this becomes now even more visible also for drug loadings further away from the PFC (i.e. the samples with 40 to 55 wt% drug). Upon storage, this phenomenon becomes even more pronounced, since now all samples between MLC and PFC (25 to 55 wt%) showed recrystallization when stored above the *T_g_* of NAP (ambient conditions) (Fig. 5). Interestingly, also the sample with 20 wt% drug loading showed some recrystallization when stored under ambient conditions, suggesting that the MLC lies below 20 wt%. This was unexpected since the experimental MLC was 20.6 wt%, however, the 95% confidence interval (19.1–22.2 wt%) indicates that the experimental MLC may indeed be below 20 wt%. An MLC below 20 wt% is also supported by the finding that the storage time (4 weeks) did not cause recrystallization of the 10 and 15 (wt%) NAP samples regardless of the storage conditions. When stored below the *T_g_* of NAP (at −80 °C), the samples were generally more stable as expected, however, the sample with a drug loading of 35 wt% recrystallized (Fig. 5), indicating that a confinement of the drug (loadings between MLC and PFC) can prolong stability below *T_g_* for some time but cannot prevent crystallization over time.

## 4 Conclusion

It was shown that the MLC can be experimentally determined for drugs that are poor glass formers under the condition that the confined drug above MLC and below PFC remains amorphous in the heat-cool-heat cycle applied in the DSC run. Using solid-state DFT and AIMD simulations, it was confirmed that the drug-monolayer binding energies are more favorable than those found in the crystalline state of the drugs, which was reflected in the physical stability of the samples below the MLC regardless of being stored below or above the *T_g_* of the drugs. On the other hand, above the MLC and below the PFC, the confined amorphous drugs are thermodynamically unstable and consequently resulted in recrystallization during storage. Nevertheless, the confinement of the amorphous drug prolonged its physical stability compared to the pure amorphous drug, which in turn allowed the experimental determination of the MLC of the poor glass former NAP.
